# Concept of Essential Medicines and Rational Use in Public Health

**DOI:** 10.4103/0970-0218.62546

**Published:** 2010-01

**Authors:** Sitanshu Sekhar Kar, Himanshu Sekhar Pradhan, Guru Prasad Mohanta

**Affiliations:** Department of Preventive & Social Medicine, JIPMER, Puducherry-6, India; 1 Specialist in Health Management, A-2/80, Janak Puri, New Delhi, India; 2 Department of Pharmacy, Annamalai University, Annamalai Nagar, Tamil Nadu, India

## Introduction

The Alma-Ata declaration during the International Conference on Primary Health Care in 1978 reaffirms that health is a fundamental human right and the attainment of the highest possible level of health is a most important worldwide social goal.(1) The Alma Ata declaration has outlined the eight essential components of primary health care and provision of essential medicines is one of them.(1) Medicines are integral parts of the health care and the modern health care is unthinkable without the availability of necessary medicines. They not only save lives and promote health, but prevent epidemics and diseases too. The medicines are undoubtedly one of the weapons of mankind to fight disease and illness. Accessibility to medicines is too the fundamental right of every person.

## Concept of Essential Medicines

World Health Organization (WHO) introduced the concept of essential medicines in 1977.(2) Essential medicines are those that satisfy the priority health care needs of the population. They are selected with due regard to public health relevance, evidence on efficacy and safety, and comparative cost-effectiveness. Essential medicines are intended to be available within the context of functioning health systems at all times in adequate amounts, in the appropriate dosage forms, with assured quality and adequate information, and at a price the individual and the community can afford. The implementation of the concept of essential medicines is intended to be flexible and adaptable to many different situations; exactly which medicines are regarded as essential remains a national responsibility. Experience has shown that careful selection of a limited range of essential medicines results in a higher quality of care, better management of medicines (including improved quality of prescribed medicines), and a more cost-effective use of available health resources. The WHO has developed the first essential medicines list in 1977 and since then the list has been revised every 2 years. The current one is the 15^th^ model list released in 2007.(2) The essential medicine list contains limited cost-effective and safe medicines, while the open pharmaceutical market is flooded with large number of medicines many of which are of doubtful value. The model list of WHO serves as a guide for the development of national and institutional essential medicine list. The concept of essential medicines has been worldwide accepted as a powerful tool to promote health equity and its impact is remarkable as the essential medicines are proved to be one of the most cost-effective elements in health care.

## Selection of Essential Medicine List

The process by which medicines are selected is critical. An essential medicines list which is imposed from above will not reflect the need of the users or be accepted by them. It is therefore very important that the process be consultative and transparent, selection criteria be explicit, selection of the medicines be linked to evidence-based standard clinical guidelines, clinical guidelines and the list be divided into levels of care, and are regularly reviewed and updated. A review of the clinical guidelines and the list should be carried out at least every second year, and their use and the impact should be monitored.(3) The process of developing a list of essential drug list was mentioned in Box 1.(4)

**Box 1:** Key factors for development of an essential medicines list

Establishing a transparent process for creating and updating the list of essential medicines, provide a voice for key stakeholders, but ensure a scientific, evidence-based process
Linkage with standard clinical guidelines and involvement of both specialists and primary care providers
Garner support from senior clinicians, academic institutions, public health professionals, professional organizations, non-governmental organizations and community members
Making the list available (essential medicines, formulary manuals and clinical guidelines) in all health care facilities/health care providers in both printed and electronic format
Consider launching of new or revised lists with the involvement of concerned government officials with adequate publicity
Making clear the specific legal or administrative authority of the essential medicines list for training, procurement, reimbursement and public information
Establishing an administrative or budgetary “safety valve” for the limited supply and use of non-listed medicines
Regular Updating of the list so that it reflects therapeutic advances and changes in cost, resistance patterns and public health relevance

## Indian Scenario

One of the vital components of the health care is medicine, as they account for a substantial part of household expenditure. The overall budget of medicines varies widely in different states in India. The expenditure pattern on medicines of the State Government shows that there are wide-ranging differences across states, from as little as less than 2% in Punjab to as much as 17% in Kerala during 2001-02.(5) The southern states such as Kerala and Tamil Nadu spend over 15% of their health budget on medicines. Many backward states, both in economic and health indicator terms, incurred the lowest expenditure on medicines. States such as Assam, Bihar, U.P., and Orissa spent about 5% or less of their health budget on medicines and medicines.(5) The Central Government's share of medicines in its total health budget is around 12%. In all, roughly 10% of the health budget goes into procuring medicines in India.(5) Even then, availability of medicines often is a big issue. The non-availability of required medicines jeopardizes the credibility of the public health system. Access to essential medicines is closely linked to health system performance and its utilization. The non-availability of essential medicines in the health facilities is not the only issue; there are problems of affordability and accessibility despite spending a large proportion of resources on medicines.(5) The availability of medicines is undermined by several factors: Poor medicine supply and distribution systems; insufficient health facilities and staff; and low investment in health, and the high cost of medicines.(5) The Essential Medicines List can help countries rationalize the purchasing and distribution of medicines, thereby reducing costs to the health system.

**Box 2:** Strategies to promote rational use of medicines

A mandated multi-disciplinary national body to coordinate medicine use policies
Clinical guidelines
Essential medicines list based on treatments of choice
Drugs and therapeutics committees in districts and hospitals
Problem-based pharmacotherapy training in undergraduate curricula
Continuing in-service medical education as a licensure requirement
Supervision, audit and feedback
Independent information on medicines
Public education about medicines
Avoidance of perverse financial incentives
Appropriate and enforced regulation
Sufficient government expenditure to ensure availability of medicines and staff

This concept of essential medicines is relatively new to India and Tamil Nadu is the first state to develop the essential medicine list as early as in 1994. Then government of Delhi too had developed its own list. The government of India and many other individual states have their own essential medicines list, and the current national list was compiled during 2003. Unfortunately, the list is not regularly up dated except for Tamil Nadu. As the list needs to be developed locally and should be based on evidence not merely on individual's experience, it is necessary first to develop clinical guidelines, called standard treatment guidelines (STG). Then based on STG the list is compiled. Delhi took the lead in developing a comprehensive Drug Policy in 1994 and was the only Indian state to have such a comprehensive policy.(6) The policy's main objective is to improve the availability and accessibility of quality essential drugs for all those in need.(6) Now many state governments too have developed STG for use within the state government health facilities. The Armed Forces Medical College (AFMC) has developed STGs for quite large number of common conditions and the treatment cost is also calculated.(7)

## Usage of Essential Medicine List

The concept of essential medicines has also been adopted by many international organizations, including the United Nations Children's Fund (UNICEF) and the Office of the United Nations High Commissioner for Refugees (UNHCR), as well as by non-governmental organizations and international non-profit supply agencies. Many of these organizations base their medicine supply system on the Model List. Lists of essential medicines also guide the procurement and supply of medicines in the public sector, schemes that reimburse medicine costs, medicine donations and local medicine production, and, furthermore, are widely used as information and education tools by health professionals. Health insurance schemes too are increasingly using national lists of essential medicines for reference purposes. The model list serves as a baseline for further modification (addition and deletion of new medicines), correct dosage strength, and form depending upon the national priority and available evidence.(8)

## Rational Use of Medicines

The selection of essential medicines is only one step towards the improvement of the quality of health care; selection needs to be followed by appropriate use. Each individual should receive the right medicine, in an adequate dose for an adequate duration, with appropriate information and follow-up treatment, and at an affordable cost. Worldwide more than 50% of all medicines are prescribed, dispensed, or sold inappropriately, while 50% of patients fail to take them correctly. Moreover, one-third of the world's population lacks access to essential medicine.(2) The situation is alarming. Unfortunately, because of inappropriate use, the effective medicines of yesterday become ineffective today. A classic example is antimicrobial medicines. Thus, in addition to achieve improved accessibility of essential medicines (availability and affordability); it is equally necessary to use the medicines appropriately, known as using rationally.

## Definition

The rational use of Medicines (RUM) is defined as “Patients receive medications appropriate to their clinical needs, in doses that meet their own individual requirements, for an adequate period of time, and at the lowest cost to them and their community.”(9) Irrational use occurs when one or more of these conditions are not met. The use of too many medicines per patients; inappropriate use of antimicrobials, often in inadequate dosage, for non-bacterial infections; over use of injections; and prescriptions not in accordance with STG; are few common types of irrational use of medicines.

The inappropriate use of medicines is widespread. It is costly and extremely harmful both to the individual and the population as a whole mainly in childhood infection and in chronic diseases like hypertension, diabetes, epilepsy, and mental disorder.(2) Increased incidence of adverse drug events and resistance is another serious issue.

## Strategies to Promote Rational Use of Medicines

The following strategies have been advocated by WHO for promoting rational use of medicines.(2)

There is 3 M concept in Rational Use of Medicines (RUM): Medicines Mean Money. Thus, RUM means less profit and income for those dealing with medicines; prescribers, and sellers. This conflict of interest is particularly relevant in our country where just only 3-5% of population are covered under any form of health insurance.(10) The medical practitioners have wide scope and responsibility too in promoting rational use of medicines for better health care. Educational strategies to health care practitioners and consumers have been proved successful model for promoting RUM. One of the educational strategies is to train the medical students of different levels on RUM. The concept and usefulness of RUM need to be the part of the curriculum. A WHO manual “Guide to Good Prescribing: a Practical Manual” is a useful publication for under graduate and post graduate students is a welcome step in this endeavor.(11)

The medical practitioners need to keep themselves updated through attending seminars, conferences, and other continuing professional development programmes. These programmes should not be supported by pharmaceutical industries, as often there is conflict of interest. They should look for independent publications or drug information centres for drug-related information, but not from the medical representatives. The hospital formulary is a good source of information. The essential medicines should be the first choice during medical practice. Finally, they should take care of their clients, the patients, by spending some time with them explaining the appropriate use of prescribed medicines. The patients should be accepted as the partner in drug therapy prescribing.

## Conclusion

The World Medicines Situation Report 2004 of the World Health Organization (WHO) pointed out that approximately 67% of the population lives without an access to essential medicines.(12) India is reckoned among the global leaders in the manufacturing of generic medicines. However, it is also held that the largest number of populace in India is living without having an access to basic medicines.(5) For meeting the requirements of medicines at reasonable prices as also for strengthening of the indigenous manufacturing capacity and capability, the Government has, over the years, formulated policies and issued drug price control orders from time to time. Currently, National Pharmaceuticals Policy, 2005 has been drafted with key objectives of price regulation of the essential medicines, availability of good quality medicine, higher investment for increased production, emphasis on drug research and development and promoting good manufacturing practice in domestic pharmaceutical companies.(13)

Government, universities, and professional associations have a critical role to play with regard to the improvement of undergraduate, postgraduate, and continuing medical education in clinical pharmacology, therapeutics, and medicines information issues. Problem-based pharmacotherapy teaching may be an effective strategy in this endeavor. We need to change our attitude and start the process of prescription audit in government and private sector and the importance of essential drug list and RUM should be emphasized in every possible forum. The essential medicine concept is relevant to other health programs as well and results not only in better use of resources but also in better practice of medicine. It addresses several other issues such as good therapeutics and reduced side-effects of medicines, and saves money for individuals, hospitals, health care providers, and the country.
